# Spending time in a forest vs. a virtual forest simulation: qualitative and quantitative effects on stress perception and psychological wellbeing in a randomized cross-over trial of highly sensitive persons

**DOI:** 10.3389/fpsyg.2026.1707766

**Published:** 2026-04-08

**Authors:** Joel Kühn, Ann-Kathrin Lederer, Carola Sättele, Roman Huber, Katja Oomen-Welke

**Affiliations:** Center for Complementary Medicine, Department of Internal Medicine II, Freiburg University Hospital, Faculty of Medicine, University of Freiburg, Freiburg, Germany

**Keywords:** 360° video, cross-over trial, forest bathing, highly sensitive persons, psychological wellbeing, qualitative interviews, stress perception, virtual reality forest

## Abstract

Forest bathing (“Shinrin-yoku”), traditionally practiced in Japan, has shown positive effects on stress perception and psychological wellbeing in highly sensitive persons (HSP), but it is unclear whether a simulation can produce the same effects. Forty-nine HSP underwent in randomized order a 40-min exposure to both an authentic forest environment and a 360° video-based virtual forest simulation using a head-mounted display, complemented by natural forest sounds and essential spruce oil diffusion. Quantitatively, wellbeing and stress reduction measured with the Change in Subjective Self-Perception as pre-specified primary outcome (CSP-14; Cohen's d: 0.931; *p* < 0.001) and secondary outcome state anxiety (STAI-S: Cohen's d: 1.5 vs. 0.275; *p* < 0.001) improved significantly more in the real forest. The forest condition also yielded greater reductions in heart rate (-7.94 vs.−3.47 bpm; Cohen's d: 0.335; *p* = 0.026) and superior improvements on Basel Well-Being Subscales vitality, intrapsychic balance, and vigilance (Cohen's d: 0.751, 0.466, 0.629; all *p* < 0.001). Furthermore, we analyzed qualitative interviews which revealed that authentic multisensory experiences in the forest significantly enhanced relaxation, reduced stress, and promoted emotional security compared to the simulation. Although the virtual environment provided temporary calming effects and facilitated reflection, technical limitations, such as visual blurring, repetitive audiovisual loops, and limited sensory diversity, considerably impaired the immersive experience. Particularly for HSP, authentic natural environments seemed to be important in our setting in achieving deeper and more sustainable psychological benefits considering the existing limitations of video-based virtual forest simulation. This study emphasizes the important value of real forest interactions in promoting psychological wellbeing and effective stress management.

**Clinical trial registration:**

https://drks.de/search/de/trial/DRKS00025421, identifier: DRKS00025421.

## 1 Introduction

Spending time in the forest for health-related purposes, commonly known as “forest bathing”, has a long tradition in Japan (“Shinrin-yoku”). In the early 2000s, the Japanese Ministry of Agriculture, Forestry and Fisheries initiated research into the health-promoting effects of spending time in the forest (Li, 2012), which various Japanese universities are currently continuing (Li, 2022). Recent systematic reviews have shown that most research on the medical use of forests still relies on pilot studies and small trials, though the number of randomized controlled trials is increasing (Kang et al., 2022; Siah et al., 2023). In addition, Antonelli et al. (2019)) have shown in a systematic review that cortisol levels were significantly lower after intervention in forest groups compared with control groups, or a significant pre-post reduction of cortisol levels was reported in the forest groups. Li (2022)) recently summarized known effects of forest bathing including stabilization of the autonomic nervous system through reduced stress hormones (decreased cortisol levels in saliva, decreased adrenaline and noradrenaline levels in urine), reduced activity of the sympathetic nervous system and an activated parasympathetic nervous system. Furthermore, forest bathing appears to reduce the activity of the prefrontal cortex, lower blood pressure and induce relaxation (Song et al., 2016; Li, 2022). Studies found a reduction in stress, tension, anxiety, anger and tiredness as well as an increase in vigor, measured with established questionnaires, after spending time in the forest in comparison to control group in urban environment for 30 min (Takayama et al., 2014), in forest vs. field environment for 1 h (Oomen-Welke et al., 2022) as well as in a systematic review of Cheng et al. (2021)). In view of these findings, forest bathing might provide an important contribution to the prevention of stress-related diseases if randomized controlled trials can strengthen the evidence for the mentioned findings.

Previous studies on quantitative effects of forest bathing often lack adequate control conditions, employ small sample sizes, and rely predominantly on self-reported outcomes, limiting the generalizability of findings (Hansen et al., 2017; Oh et al., 2017). The heterogeneity of intervention protocols and outcome measures further complicates cross-study comparisons (Wen et al., 2019). These limitations underscore the need for rigorous controlled study designs. Additional qualitative explorations are helpful to capture experiential dimensions that standardized instruments may not fully assess and might help to evaluate mechanisms of action.

The mechanism of action of spending time in the forest on human health has not yet been conclusively clarified. Qualitative interviews suggest that sensory perceptions mediate the relaxing and restorative effect of spending time in the forest (Gallis et al., 2012; Oomen-Welke et al., 2023). Such sensory perceptions may be sounds (e.g. rustling leaves, singing birds, rushing water) smells (e.g., essential oils emitted by trees and the smell of wood and forest floor) and visual impressions (e.g., the play of light and shadow that characterizes the forest atmosphere). The height of the trees in a forest has been shown to provide a sense of security (Oomen-Welke et al., 2022, 2023). These findings may be explained by Ulrich's theoretical framework stress recovery theory (SRT) that emphasizes the rapid physiological stress reduction through aesthetically pleasing natural stimuli. Additionally, Kaplan's attention restoration theory (ART) focuses on the recovery of cognitive resources, particularly “directed attention,” through exposure to fascinating yet undemanding natural environments (Ulrich, 1983; Kaplan, 1995).

Aside from the fact that exposure to nature can improve mental health and reduce stress, recent findings demonstrate that even virtual exposure to natural environments significantly reduces self-reported pain and discomfort as well as having positive effects on relaxation and wellbeing (Hejtmánek et al., 2022; Ortega et al., 2024; Masters et al., 2025). Neuroimaging data have further indicated that images of virtual nature environments can induce genuine analgesic effects by modulating nociception-related neural processes, such as reducing activation in the thalamus, secondary somatosensory cortex, and posterior insula which may be indicative of a more generalized stress regulatory response (Steininger et al., 2025).

Virtual nature exposure encompasses a spectrum of technological implementations with varying degrees of immersion and interactivity. At the lower end, static nature imagery (photographs, videos on conventional screens) provides only visual stimulation with limited engagement. Mid-level approaches include 360° video environments presented through head-mounted displays (HMDs), which expand the visual field and enable naturalistic head-tracked viewing but remain fundamentally passive and non-interactive. At the upper end, fully interactive virtual reality (VR) systems combine stereoscopic 3D rendering, spatial audio, haptic feedback, and real-time environmental responsiveness, approaching the multisensory variety of authentic nature exposure (Slater and Sanchez-Vives, 2016; Cummings and Bailenson, 2016). These distinctions are critical for interpreting study outcomes, as immersion level directly influences presence, embodiment, and psychological response (Riva et al., 2007).

To our knowledge, no randomized controlled cross-over study has directly compared *in-situ* forest exposure with a matched multimodal virtual forest within the same sample. If the sensory perceptions play the key role for the positive health effects, a simulation should be effective as well. Therefore, we implemented a 360° video-based virtual forest simulation using head-mounted displays (HMDs) to simulate the visual impressions of the forest through a monoscopic 360° video recorded at the same site as the in-situ condition. Acoustic impressions were simulated by synchronized playback of natural forest soundscapes recorded on-site, and olfactory cues through evaporative diffusion of coniferous essential oils. This mid-level immersion approach (passive, video-based, and multimodal with visual, auditory and olfactory stimuli) was selected to balance ecological validity, technical feasibility, and participant comfort, while remaining distinct from fully interactive VR systems with stereoscopic rendering and haptic feedback (Slater and Sanchez-Vives, 2016). Slopes, unevenness, the structure of the ground and haptic impressions are more complex to simulate. A simulation while being in a room cannot perfectly replicate these sensations. Furthermore, its implementation in daily life is significantly more straightforward, especially for those who lack ready access to forests. If the simulation were as effective in reducing stress as a stay in the forest, this would have significant implications for health promotion and disease management, as it would represent a cost-effective and widely accessible alternative to spending time in natural environments.

Given these findings and in view of the increase in mental health problems and new stress, especially in young adults after the pandemic (Tian et al., 2022; Bie et al., 2024), it would be beneficial to have suitable options for improving mental health through accessible, evidence-based interventions which should be easy to access. Therefore, we intensified our research regarding spending time in a forest, or alternatively, virtual nature environment on stress perception and psychological wellbeing. To address this need and the current evidence gap, this randomized, controlled cross-over trial directly compares outcomes after authentic *in-situ* forest exposure with those after a matched 360° video-based virtual forest simulation delivered via HMD. To enhance sensitivity to subtle environmental differences in stress perception and psychological wellbeing, we recruited highly sensitive persons (HSP) as the study population.

HSP react intensely to subtle stimuli that others may not even be aware of. These stimuli may include the behavior or mood of other people, the influence of the media, the consumption of caffeine or certain medications, physical discomfort, and hunger (Vander Elst et al., 2019). They experience their emotional reactions to both positive and negative stimuli to a greater extent and process them at a deeper level, which can lead to a longer reaction time (Aron et al., 2012), but also to more intense feelings and easier emotional excitability (Vander Elst et al., 2019). Due to their low perception threshold, they are often overwhelmed by stimuli, experience stress more quickly than others, and are more easily emotionally aroused (Aron and Aron, 1997; Hinterberger et al., 2019). One strategy for avoiding negative feelings can be shyness, introversion and social withdrawal (Aron et al., 2012).

Due to their low threshold and strong reactions to minor stimuli, HSP are likely to react intensely to a changed environment. We, therefore, assumed that HSP would depict the differences between spending time in the forest and forest simulation sensitively. Recent research demonstrates that HSP exhibit significantly stronger nature connectedness (Setti et al., 2022; Holzer et al., 2024) and respond particularly well to nature-based interventions (Cadogan et al., 2023). Our primary objective was to test whether a real forest exposure elicits greater immediate improvements in subjective state than a matched video-based forest simulation, operationalized as higher post-exposure CSP-14 total and subscale scores in HSP. Secondarily, we hypothesized that the forest would produce larger pre–post gains in mood (Basel Mood Scale), greater reductions in state anxiety (STAI-S), and more favorable changes in physiological markers (heart rate, blood pressure, and the pulse-respiration quotient) relative to the simulation. Furthermore, we conducted semi-structured interviews to identify and evaluate differences between the forest and the forest simulation conditions.

## 2 Materials and methods

### 2.1 Trial design

We designed a randomized, controlled, monocentric study employing a cross-over methodology. The study comprised two intervention arms: participants in each arm underwent both a 40-min exposure to a natural forest environment and a 40-min session in a simulated forest environment, with the order of interventions counterbalanced across groups. We assigned participants to the intervention arms in a 1:1 ratio. The study protocol remained consistent throughout the trial. Each intervention group included a maximum of ten participants to avoid mutual disturbance among the participants.

### 2.2 In- and exclusion criteria and recruitment procedure

We identified HSP using the SV12 scale and included those with a total score of items >18, indicating over average sensitivity with an age between 18–70 years. Exclusion criteria comprised severe physical or mental illness, substance-induced disorders, language barriers, pregnancy, and breastfeeding. We defined severe mental illness using the symptom rating scale (ISR) from the 10^th^ edition of the International Classification of Diseases (ICD-10), excluding individuals with a total ISR score exceeding 0.9 (Tritt et al., 2008). We recruited via social media and local newspapers. Two medical students screened interested people within a comprehensive phone interview. When the telephone screening yielded insufficient information or doubts regarding eligibility, a physician qualified in psychotherapy conducted an in-person assessment and provided final evaluation of suitability. Written informed consent was obtained when screening procedure met all in- and exclusion criteria and was collected via E-mail or before the first intervention started.

### 2.3 Quantitative outcomes and measures

The primary outcome was the CSP-14, a 14-item feedback instrument designed to capture immediate changes in physical (5 items), emotional (6 items), and mental (3 items) states after an intervention. Each item was rated on a bipolar scale from−3 to +3, with higher values indicating improvement. Items were aggregated into three subscales according to the published structure: Integration (physical items 1–4, emotional items 5–6, mental item 1), Balance (emotional items 1–2), and Vitality (physical item 5; emotional items 3–4; mental items 2–3). The primary endpoint was the within-participant comparison of the CSP-14 total score between the real forest and the forest simulation conditions; the three CSP-14 subscales were analyzed as pre-specified secondary endpoints.

Secondary self-report outcomes included the BMS and the STAI-S. The BMS is a 16-item instrument rated on seven-point bipolar scales and summarized into four subscales-intrapsychic balance, vitality, social extroversion, and vigilance where higher scores reflecting better mood. The STAI-S includes 20 items rated from 1 to 4; 10 positively worded items were reverse-scored per manual, and item scores were summed to yield a total between 20 and 80, with higher scores indicating greater state anxiety.

Physiological outcomes included heart rate, blood pressure, respiratory rate, and the pulse-respiration quotient (PRQ). Heart rate and blood pressure were measured with a wrist blood-pressure device after a minimum of 5 min of seated rest. Respiratory rate was assessed by visual counting of thoracic movements. The PRQ was computed as the ratio of heart rate to respiratory rate, providing a composite index of cardiorespiratory regulation.

Quantitative assessments followed a standardized pre-post protocol at each session in both conditions. Before the 40-min exposure (pre, t1), participants completed the BMS and STAI-S and then, after at least 5 min of seated rest, underwent measurements of blood pressure, heart rate, and respiratory rate. Immediately after the 40-min exposure (post, t2), participants again sat quietly for at least 5 min prior to repeat physiological measurements and then completed the CSP-14, BMS, and STAI-S.

### 2.4 Semi-structured interviews

We audio-recorded all interviews using a Tascam DR-40 device from TEAC Europe GmbH, with interview durations ranging from 5 to 24 min. We developed the interview guide by a systematic approach for developing scientific interview guides (Helfferich, 2019). In a first step, we collected as many questions as possible in an open brainstorming. We then checked the questions for suitability, summarized them into groups and formulated broad and open questions that included all the detail-questions of these groups. We used these open questions as guiding questions in the interview guide. Additionally, we included specific, pre-formulated questions to elicit detailed information from the interviewees (Helfferich, 2011; Moser and Korstjens, 2018). Prior to data collection, interviewers received training through role simulations to minimize interviewer bias and avoid influencing participants with suggestive questions.

The key interview questions included:

- “You have just spent 40 min in the forest/forest simulation. How did you experience it?”
- “How are you feeling now?”
- “What appeals to you in general about the forest/forest simulation?”
- “Wonder question: What would the ideal forest/forest simulation has to look like for you to feel particularly comfortable there?”

We conducted semi-structured interviews with two randomly selected participants per intervention session (see Sections 2.6–2.7), yielding 48 interviews in total. The primary objective of the interviews was to capture participants' sensations, emotions, and potential triggers for these feelings, as well as to assess changes in their mental state over time.

### 2.5 Intervention

All participants except one completed both interventions in a counterbalanced order. Group A first experienced the natural forest environment, followed by the simulated forest environment, whereas Group B underwent the simulated environment before the natural forest exposure. Every group was limited to a maximum of 10 participants. A 1-week washout period separated the two interventions to minimize carryover effects.

The natural forest intervention took place in the Freiburg city forest, a typical mixed forest representative of southern Germany and the Black Forest region. The site, located outside the urban area yet easily accessible, occasionally exposed participants to visual and auditory stimuli from passersby and, on rare occasions, to noise from forestry activities. Researchers documented these disturbances in a dedicated log. After participants have traveled independently and arrived at the meeting point (Wonnhaldestraße 6, 79100 Freiburg, Germany), researchers welcomed participants and conducted baseline assessments, including measurements of blood pressure, heart rate, respiratory rate, and administration of the State-Trait Anxiety Inventory (STAI) and Basel Mood Scale (BMS) questionnaires after they were seated for 5 min. We then guided the participants silently to the intervention site, approximately 400 meters away, situated within a demarcated 30 ^*^ 30 meters area off established paths. The intervention site was a mixed forest in a hilly area with single trees up to over 100 years of age and above 20 meters height. The ground between the trees was covered with grass or herbs, moss or without visible vegetation (Supplementary Figure S1). The participants chose a place within this area where they sat on the ground, a blanket or a tree stump. Interventions occurred during late summer (June to July 2023), with ambient temperatures ranging from 21 to 30 °C (mean: 25.2 °C), relative humidity between 45% and 75%, and weather conditions varying from cloudy to sunny. After the stay in the forest, blood pressure was measured after 5 min of sitting, and participants completed the CSP-14, BMS, and STAI questionnaires. Following each 40-min forest exposure, researchers conducted semi-structured interviews with participants during the return walk to the meeting point.

Participants assigned to the virtual reality (VR) simulation attended the intervention in a seminar room at the University Medical Center Freiburg. Participants viewed a 6.5-min video clip as a 360° VR monoscopic video (resolution: 6,144 × 3,072, video data rate: 122.5 Mbps, 24 Hz) of the forest using a Pico 4 VR headset from Pico Technology Co. Ltd. (resolution: 4,320 × 2,160; 2,160 × 2,160 per eye; 1,200 PPI), which translated head movements into corresponding changes in the visual field. To ensure safety, participants remained seated throughout the session. The headset delivered a natural audio soundtrack recorded in the actual forest, and the video, filmed in the same forest location in early June, played in a continuous loop for the duration of the intervention. Additionally, two nebulisers in the room dispersed essential spruce needle oil to the feeling of a natural environment. Environmental conditions during the VR intervention mirrored those of the forest, with temperatures between 21 and 30 °C (mean: 26.5 °C) and relative humidity from 40% to 70%. While the temperature was above 26 °C (7 out of 12 interventions) two fans were used to get air circulation in the room. Questionnaires, interviews and physiological measurements were performed analogous to the forest intervention group.

### 2.6 Sample size

The primary aim of this study was to compare the effects of real forest exposure and video-based forest simulation on emotional, mental, and bodily wellbeing in highly sensitive individuals (SV12), measured by the total CSP-14 score. The study incorporated both qualitative and quantitative outcome measures. We calculated the sample size according to the primary outcome. As published data on the standard deviation or variance for the CSP-14 questionnaire are unavailable, we estimated the required sample size using the standardized effect size (Cohen's d). Following conservative planning principles for exploratory research (Cohen, 2013), we assumed a medium effect size (d = 0.5), a statistical power of 80%, an alpha level of 0.05, and a two-sided paired *t*-test within the cross-over design. Based on these parameters, a minimum of 34 participants was required. Assuming no carry-over effects and no interaction between participants, groups, or periods, and accounting for an anticipated dropout rate of 15%, we aimed to recruit at least 40 participants.

For the qualitative part, we selected two participants through a randomization list from each intervention group for semi-structured interviews. With a total of 24 intervention groups, this approach yielded 48 interviews (24 following the forest intervention and 24 following the simulation).

### 2.7 Randomization and allocation concealment

An independent researcher not involved in enrolment or assessment randomized the participants into study arm group A and B using a computer-based random number generator (http://www.randomization.com) with a block length of ten. For the qualitative component, a separate computer-generated randomization list was created prior to the first intervention period, determining the sequential order for interview selection within each time slot. For each intervention, two participants were also randomly selected for an interview, as well as two alternates. For logistical reasons (different meeting points), the coordinator e-mailed the participant the meeting point the day before the first session, which implicitly revealed the first-period condition. Blinding of participants and field staff after assignment was not feasible due to the nature of the interventions.

The first two participants according to the randomization sequence who were present and willing to participate were invited for semi-structured interviews following both the first and second intervention, maintaining the cross-over design structure. In cases where a primary participant was unavailable or had dropped out, the first of the two pre-selected alternates was invited according to the randomization sequence. This procedure ensured balanced representation across intervention sequences while preserving randomization integrity and minimizing selection bias in the qualitative sample.

### 2.8 Data management and statistical analysis

The primary analysis followed the intention-to-treat principle and included all randomized participants who initiated an intervention. A per-protocol sensitivity analysis included participants who completed at least 75% of the intervention. Missing values were examined for their pattern and imputed using the series-mean procedure implemented in SPSS version 29.

Data distributions were assessed with Kolmogorov-Smirnov tests. To evaluate comparability of groups, baseline continuous variables were compared using independent samples *t*-tests, and baseline categorical variables using chi-square tests. All hypothesis tests were two-sided with an alpha level of 0.05. Effect sizes were quantified as Cohen's d and were interpreted as small (0.2–0.4), medium (0.5–0.7), and large (≥0.8–1). The analysis was performed following the recommended methodology for crossover studies (Wellek and Blettner, 2012).

For the primary endpoint, we compared the CSP-14 total score between the real forest and VR forest conditions within participants using paired *t-*tests. Prespecified analyses of the CSP-14 subscales (Integration, Balance, Vitality) used the same approach.

For secondary self-report outcomes (BMS, STAI-S) and physiological parameters (heart rate, blood pressure, PRQ), we performed within-condition pre-post comparisons (t2–t1) using paired *t-*tests. To compare conditions, we computed individual change scores (post-pre) for each session and tested the difference of these change scores between the real forest and VR sessions with paired *t-*tests. All secondary analyses were adjusted for multiple comparisons using the Holm-Bonferroni correction.

To evaluate potential carryover or period effects inherent to the cross-over design, we implemented a 1-week washout and conducted a priori order checks. Specifically, we compared condition-specific outcomes between the “forest-first” and “simulation-first” groups using independent-samples *t-*tests; non-significant tests were interpreted as absence of meaningful carryover.

Exploratory correlations were planned to examine associations between post-intervention CSP-14 total scores and secondary outcomes (BMS total, STAI-S, blood pressure, and PRQ). After confirming distributional assumptions, we estimated Pearson correlation coefficients and reported r and two-sided *p*-values.

All analyses were pre-specified for the quantitative component of the study and were applied identically across both intervention conditions.

### 2.9 Transcription and evaluation methods

We transcribed all interviews automatically using GoSpeech software (GRUNDIG Business Systems GmbH & Co. KG) in accordance with GDPR requirements. Subsequently, we manually reviewed and corrected the transcripts to ensure high accuracy and quality. During the transcription process, we pseudonymized all personal identifiers, such as interviewee gender, and replaced them with anonymized participant numbers to safeguard participant confidentiality. We stored the finalized transcripts, complete with time stamps, in Word format (.docx) and archived them in a tamper-proof database.

For qualitative analysis, we utilized MAXQDA 2024 software and adopted a combined methodological approach, integrating grounded theory and qualitative content analysis as described by Kruse (2015)) and (Kuckartz and Rädiker 2024). We established two principal categories: one focused on sensory perceptions, and the other on emotions, feelings, and thoughts. Each category comprised specific codes identified during the analysis. We conducted the data analysis both inductively and deductively. Deductively, we applied pre-existing categories and codes from prior research by Oomen-Welke et al. (2023)) while inductively, we developed new categories emerging from the data.

Two independent researchers coded the interview texts in parallel to enhance objectivity and reliability. After coding 25% of the data, we compared the codebooks generated by both researchers, discussed any discrepancies, and discussed any discrepancies to establish conceptual alignment. Both researchers then continued coding independently, allowing for individual analytical development within a shared conceptual framework. Following independent coding, discrepancies were resolved through iterative discussion and consensus-building. The resulting consolidated codebook integrated both researchers' analytical perspectives. In accordance with the principle of theoretical saturation as outlined by Strauss et al. (2012)), categorization was considered complete when no substantially new thematic aspects were identified in subsequent interviews, thereby precluding the formation of additional subcategories. Following a thorough analysis of approximately two-thirds of the material (32 of the 48 interviews), it was determined that only singular, person-specific characteristics remained that could be assigned to the existing subcategories.

## 3 Results

**Figure 1:**
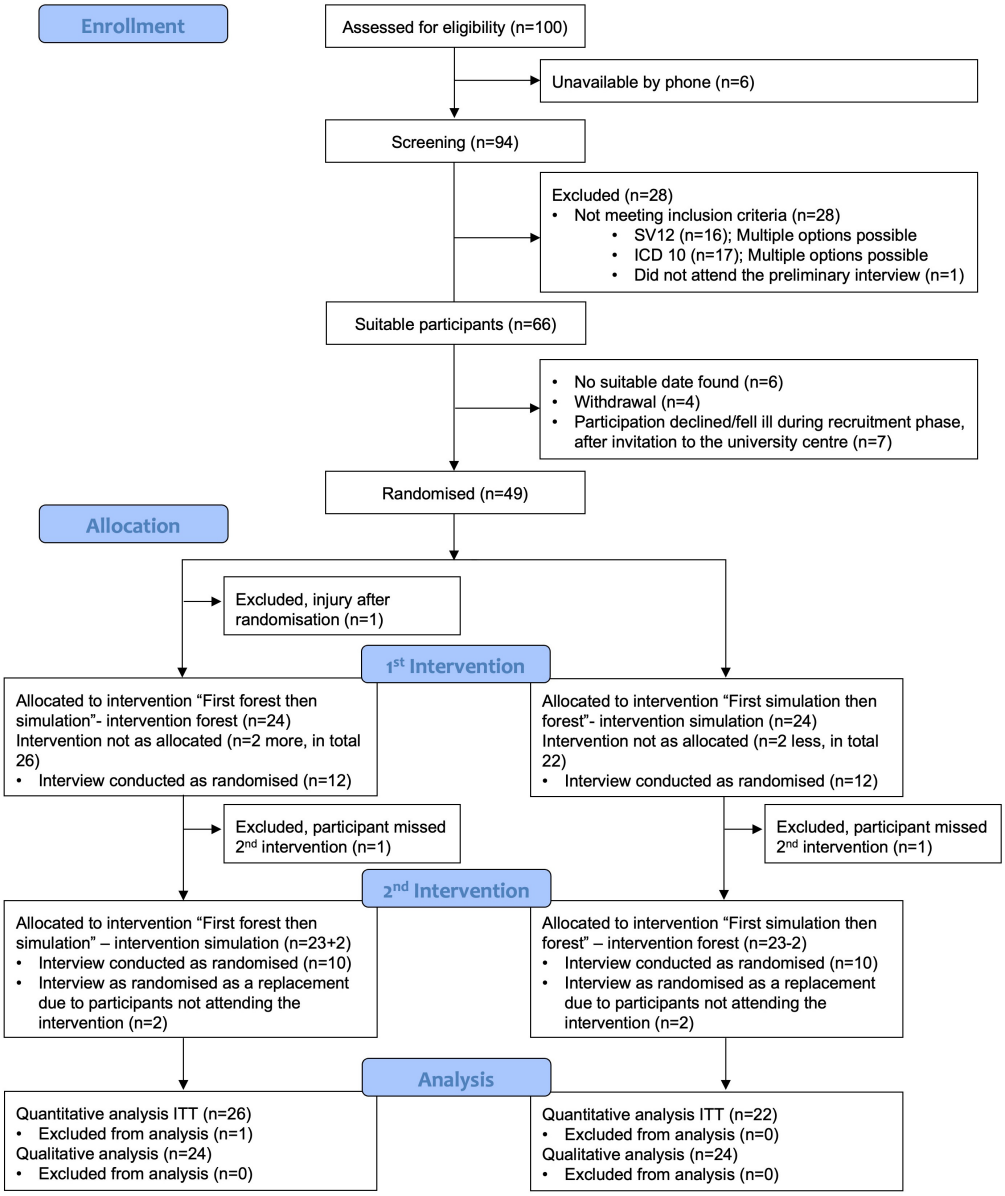
CONSORT flow diagram of participant enrollment, allocation, follow-up and analysis.

We randomized 49 participants to two sequences (forest → simulation: *n* = 25; simulation → forest: *n* = 24). The intention-to-treat (ITT) population comprised 48 participants. One randomized participant was injured before the first session and did not receive any intervention. Two participants completed only the first period due to illness, childcare and therefore contributed their first-period data to the ITT analyses but were excluded from the per-protocol (PP) set, which required completion of ≥75% of both sessions. Characteristics of the groups are shown in Table 1, the flow of the study with enrollment, allocation, follow-up, and analysis of the participants is shown in Figure 1. Most of the participants were females (81.25%), mean age was 32 years. There were no significant differences in baseline variables between the participants.

**Table 1:** Group characteristics intention to treat.

Group characteristics	Forest/simulation	Simulation/forest	Total
Participants total/both interventions (n)	26	22	48/46
Sex (f/m)	22/4	17/5	39/9
Age (years)	32.7 ± 15.0	32.0 ± 11.2	32.4 ± 13.3
SV12 total sensitivity	20.4 ± 1.4	20.7 ± 1.6	20.5 ± 1.5
ISR total score	0.3 ± 0.3	0.3 ± 0.3	0.3 ± 0.3
ISR symptom load (none/small/medium/heavy)	21/0/5/0	17/1/4/0	38/1/9/0
Employed (e/s/ue/rt)	6/17/2/1	13/9/0/0	19/26/2/1
Interview duration forest (Min/Max/Med) [min]			10/23/22.5
Interview duration simulation (Min/Max/Med) [min]			5/24/16.5

f, female; m, male; e, employed; s, student; ue, unemployed; rt, retired; SV12 16–18 average sensitivity, max. score 24; ISR ICD-10 symptom rating total score (< 0.5 no psychiatric disease).

Regarding the inclusion criterion of high sensitivity (SV12 score >18), the collective had an average sensitivity score of 20.5 ± 1.5. This value is above the threshold of 18 points, confirming the targeted selection of highly sensitive individuals.

The average psychological symptom burden, as measured by the ISR total score, was 0.3 ± 0.3 points. These values were lower than the threshold value of 0.5, indicating no suspicion of psychological symptom burden according to the ICD-10 criteria. The majority of the participants (79%) had no symptom burden.

### 3.1 Quantitative results

#### 3.1.1 Primary target parameter: change in subjective self-perception

**Figure 2:**
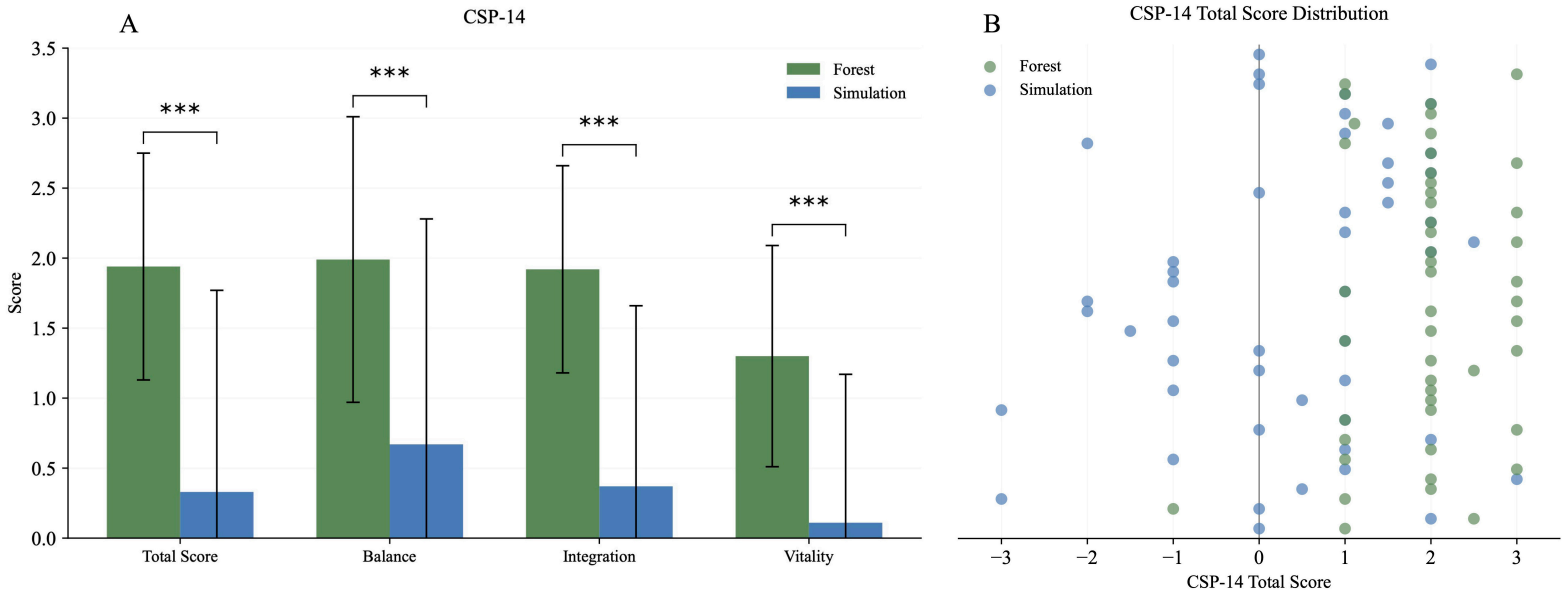
CSP-14. **(A)** Bar chart showing the CSP-14 scales “Total Score”, “Balance”, “Integration”, and “Vitality” for two conditions: Forest (green) and Simulation (blue). Bars represent mean values, and error bars indicate standard deviations. Asterisks (^***^) denote statistically significant differences between conditions (*p* < 0.001). **(B)** Scatterplot showing individual overall CSP-14 scores. Each point represents one participant. The plot compares responses between the Forest (green dots) and the Simulation (blue dots).

The CSP-14 scores were recorded immediately after the forest and simulation stay. Higher values reflected stronger positive changes. The ITT analysis showed consistently higher values in favor of the forest stay compared to the simulation (all comparisons two-sided, *p* < 0.001; large effects). The results are shown in Table 2 and Figure 2.

**Table 2:** Change in subjective self-perception (CSP-14) questionnaire.

	Average MW	Standard deviation SD	Significance level *p*-value	Effect size Cohen's d	T	df
Total score CSP-14						
Forest	1.94	0.81	< 0.001^***^	0.931	6.381	46
Simulation	0.33	1.44				
Balance						
Forest	1.99	1.02	< 0.001^***^	0.741	5.079	46
Simulation	0.67	1.61				
Integration						
Forest	1.92	0.74	< 0.001^***^	0.978	6.704	46
Simulation	0.37	1.29				
Vitality						
Forest	1.3	0.79	< 0.001^***^	0.871	5.971	46
Simulation	0.11	1.06				

Comparison between forest and simulation in the ITT population. *p*, significance level ^***^p < 0.001, a higher total score corresponds to a more positive evaluation; df, degrees of freedom.

#### 3.1.2 Secondary target parameters

**Figure 3:**
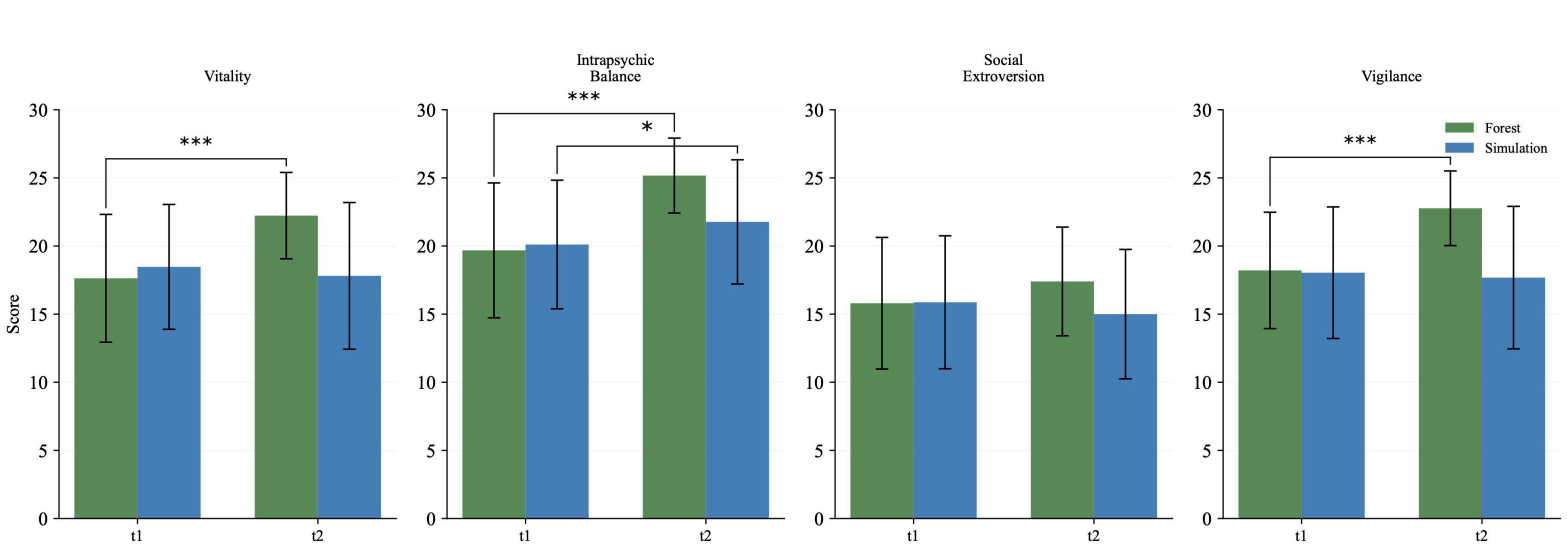
Basel Well-Being Scale. ***p < 0.001, **p < 0.01, *p < 0.05.

The Basel Well-Being Scale was assessed before (t1) and after (t2) each condition. In the ITT analysis, significant improvements were observed in the forest in the dimensions of vitality, intrapsychic balance, social extroversion and vigilance, while the simulation showed no or only minor changes. A direct comparison of the change values (Δ = t2-t1) revealed significant advantages in favor of the forest stay in all four dimensions (*p* < 0.05) whereas three dimensions showing *p* < 0.001 and small to medium effect sizes (Cohen's d: 0.751, 0.629, 0.466; see Table 3 and Figure 3).

**Table 3:** Basel Well-Being Scale. Comparison between forest and simulation in the ITT population.

	MW	SD	Significance level *p*-value	Cohen's d	Δ Diff	Significance level *p*-value 2	Cohen's d 2
Vitality							
Forest t1	17.63	4.69	< 0.001^***^	0.962	−4.6	< 0.001^***^	0.751
Forest t2	22.23	3.17					
Simulation t1	18.47	4.58	0.397	0.125	0.66		
Simulation t2	17.81	5.38					
Intrapsychic balance							
Forest t1	19.68	4.95	< 0.001^***^	1.092	−5.5	< 0.001^***^	0.466
Forest t2	25.17	2.75					
Simulation t1	20.11	4.72	0.048^*^	0.296	−1.66		
Simulation t2	21.77	4.56					
Social extroversion							
Forest t1	15.8	4.83	0.052	0.291	−1.6	0.031^*^	0.324
Forest t2	17.4	3.99					
Simulation t1	15.87	4.88	0.191	0.194	0.87		
Simulation t2	15	4.75					
Vigilance							
Forest t1	18.21	4.27	< 0.001^***^	1.059	−4.55	< 0.001^***^	0.629
Forest t2	22.77	2.74					
Simulation t1	18.04	4.83	0.683	0.06	0.36		
Simulation t2	17.68	5.23					

MW, mean value; SD, standard deviation; *p* = significance level of the paired *t*-test comparison between t1 and t2; ^***^p < 0.001, ^**^p < 0.01, ^*^p < 0.05; t1, before the intervention; t2, after the intervention; d, Cohen's d effect size; Diff, difference t2 - t1; p2, significance level of the paired *t*-test Comparison between forest and simulation; d2, Cohen's d effect size Comparison between forest and simulation: the higher the values, the higher the state.

**Figure 4:**
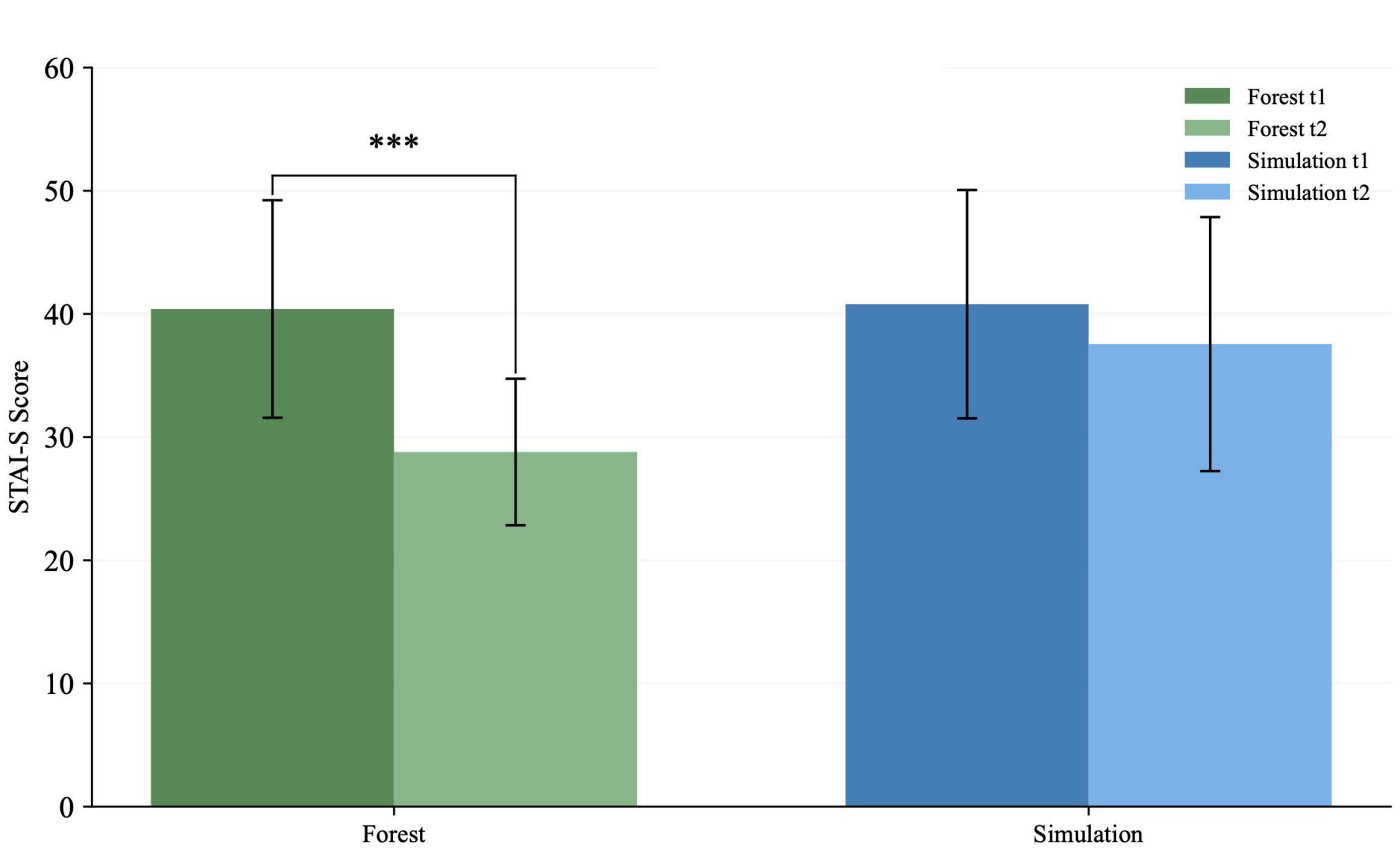
STAI-S score. ***p < 0.001.

State anxiety decreased significantly after the forest exposure by 28.74% (from 40.40 to 28.79 points; *p* < 0.001; Cohen's d = 1.50, large). During the simulation, the pre–post decrease was smaller (-3.24 points;−7.95%; *p* = 0.065; Cohen's d = 0.28, small). The between-condition difference (forest vs. simulation) was highly significant (*p* < 0.001) with a moderate effect (Cohen's d = 0.53). Notably, the forest effect was about 5.5 times larger than the simulation effect (see Table 4, Figure 4).

**Table 4:** State trait anxiety inventory comparison between forest and simulation of the entire collective (ITT).

	MW	SD	Significance level *p*-value	Cohen's d	Δ Diff	Significance level *p*-value 2	Cohen's d 2
STAI							
Forest t1	40.4	8.83	< 0.001^***^	1.5	11.62	< 0.001^***^	0.528
Forest t2	28.79	5.95					
Simulation t1	40.79	9.27	0.065	0.275	3.23		
Simulation t2	37.55	10.31					

MW, mean value; SD, standard deviation; *p* = significance level of the paired *t*-test comparison between t1 and t2, ^***^p < 0.001, ^**^p < 0.01, ^*^p < 0.05; t1, before the intervention; t2, after the intervention; d, Cohen's d effect size; Diff, difference t2-t1; p2, significance level of the paired *t*-test Comparison between forest and simulation; d2, Cohen's d effect size Comparison between forest and simulation: the higher the values, the higher the current state anxiety.

In the ITT analysis, heart rate decreased both after the forest visit (77.70 ± 10.64 to 69.77 ± 11.26 bpm; Δ = −7.94; *p* < 0.001; Cohen's d = 0.941) and after the simulation (76.53 ± 12.02 to 73.06 ± 11.97; Δ = −3.47; *p* = 0.009; d = 0.937). The reduction was significantly more pronounced in the forest than in the simulation (comparison of Δ values: *p* = 0.026; d = 0.335).

There were no pre-post changes in systolic and diastolic blood pressure (forest: systolic 121 ± 9 to 123 ± 14.5 mmHg; diastolic 76 ± 10 to 76 ± 13 mmHg; simulation: systolic 120 ± 10 to 118 ± 12 mmHg; diastolic 75 ± 11 to 74 ± 11 mmHg).

The PRQ did not change significantly in any condition (forest: 5.36 ± 1.50 to 5.25 ± 1.20; simulation: 5.09 ± 1.37 to 5.25 ± 1.42).

#### 3.1.3 Side effects

No side effects related to the interventions were reported. Two participants did not attend the second session due to time constraints or illness; there was no connection with the intervention.

### 3.2 Qualitative results

In total, we conducted 48 interviews (see Figure 1 and Supplementary Table S1).

To assess intercoder reliability, we systematically compared coding decisions for eight main thematic categories across all interviews. Agreement was achieved if the same code was used by both researchers per interview. Overall percentage agreement across categories used more than 40 times in all interviews was 78.5% (223 agreements of 284 coding decisions), indicating substantial consistency in code application. Agreement rates varied by category complexity: sensory perception codes showed high agreement (73.2%−93.5%, M = 86.5%), while emotional categories showed moderate agreement (35.0%−73.1%, M = 56.0%). These differences reflect the inherently more interpretive nature of emotional coding compared to concrete sensory descriptions. The highest agreement was observed for the central thematic category “Calmness/Stress” (93.5 %), which represented our most frequently applied code (n = 269 segments, see Supplementary Table S2).

#### 3.2.1 Sensory perceptions in forest and simulation

##### 3.2.1.1 Haptic interaction

The experiences of the participants during the forest simulation encompassed almost all sensory modalities except for the sense of taste. A major limitation of the simulation was the lack of opportunities for haptic interaction with the environment. The discrepancy between visually perceived but not physically experienced tactile stimuli diminished the sense of immersion. Participants emphasized that haptic sensations were essential for a “more intense” and authentic experience:

“*This walking around, touching, touching, connecting with nature. Of course, that's missing in a simulation like this.” 99, simulation, pos. 21*

Participants described how tactile experiences in the real forest, such as feeling moss, bark or the forest floor, helped them to ground themselves and let go of intrusive thoughts:

“*Powerful and refueling, bringing you back from your head to your center. [...] exploring textures. I definitely find that this brings me like back to the feet.” 29, forest, pos.7–19*

“*I actually found it really nice to be able to play with the different tactile sensations, [...] I had the feeling that my senses were very focused or very sharpened.” 56, forest, pos. 35*

The restrictions on movement and the requirement to remain seated during the simulation did not correspond to participants' expectations of a forest experience, and several described the simulation as restrictive (34, 95, 98):

“*I had the feeling that I couldn't let myself go completely because I was sitting on a chair. [...] This complete letting go, like you would be sitting on a bench in the forest [...] I don't think it came across quite like that.” 90, simulation, pos. 35*

In contrast, the freedom of movement in the real forest was perceived as liberating:

“*I had the feeling that I was so free. [...] Yes, somehow I was able to move around like that.” 36, forest, pos. 15–19*

Air quality was another sensory aspect that could not be authentically simulated. The fresh air in the forest was described as invigorating and health-promoting, a quality that was notably absent in the simulation:

“*The first time [in the forest], I found it incredibly pleasant to feel the fresh forest air on my skin. I missed that so much.” 95, forest, pos. 8–12*

“*It was like drinking spring water with every breath. It had such a really refreshing character.”95, forest, pos. 16*

After the forest intervention participants reported to perceive the uneven, soft and natural forest floor as much more pleasant and varied compared to hard, artificial surfaces such as asphalt. This difference reduced the monotony when walking and was described as refreshing. Some participants reported that touching objects such as moss, leaves or acorns and the perception of this tactile sensation reinforced the connection to nature and created a more intense experience in the intervention (*n* = 10). Haptic experience was important to create a sensory connection with the forest and its surroundings. Touching led to a more intense, personal experience, increasing awareness of the surroundings and participants own impact on nature and vice versa.

“*Yes, for example the acorns that I now have in my hand. Yes, [...] in connection with looking at it, feeling it, it has already triggered a certain connection in me, I would say, in the moment you touch it.” 34, forest, pos. 70*

##### 3.2.1.2 Acoustic perception

The natural sounds recorded in the forest such as birdsong, the rustling of leaves and the rippling of water triggered positive emotions. These sounds contributed to relaxation and a sense of wellbeing, as they are often associated with positive experiences such as familiarity and security. Twenty three participants explicitly described natural sounds like birdsong, leaf rustling, and flowing water as calming and emotionally positive.

“*I always find the rustling of the leaves quite pleasant. [...] Yes, relaxing.” 39, forest, pos. 79–83*

In the simulation, nature sounds had a calming effect and strengthened the illusion of an authentic experience of nature. This was explicitly confirmed in 12 interviews (*n* = 12), in which participants described the audio as relaxing, supportive of immersion, and enhancing authenticity.

Some participants showed an emotional reaction triggered by the sound of the simulation (*n* = 10). The sounds promoted the evocation of positive memories. Overall, the acoustic component had a significant influence on the overall perception of the simulation.

“*I myself want to offer relaxation courses myself soon and I also want forest sounds or something else. [...] I'm also convinced that the sounds of nature calm you down.” 23, simulation, pos. 19*

“*And I also liked the splashing, because I imagined a stream flowing along somewhere.” 63, simulation, pos. 23*

The sounds of nature contributed to calming down and could also revive memories and associated emotional connections in the participants, which were often associated with their childhood, family or friends. As a result, a feeling of security was perceived, which contributes to a sense of wellbeing in the forest.

“*For example, I found the water very calming. You can hear the water flowing.” 67, forest, pos. 51*

“*Yes, I somehow associate it very quickly with my family, because I've been around a lot of people or something. My father is also a forester, so I somehow have a connection to it. That's why it's always been a feel-good place for me and it makes me feel right at home.” 28, forest, pos. 79*

Several participants (*n* = 14) reported reduced immersion due to short, repeating audio loops or disturbing external noises such as trains, helicopters, or construction. Others criticized the lack of diversity compared with the dynamic soundscape of the real forest (e.g., wind, insects). These factors partially diminished the otherwise calming and authentic effect of the sounds in the simulation. Overall, however, the acoustic input was consistently described as central to relaxation and authenticity in both conditions.

##### 3.2.1.3 Olfactory perceptions

We were not able to simulate the “*fresh smell of the forest”* of “*humid earth” (56)*, “*moss and rotting wood” (90)*, “*forest soil and leaves” (98)*. Some described the scent of spruce needle, which was diffused by two diffusers during the simulation as too weak while it was too intense for others.

“*And also this artificial smell was not completely the forest for me, it smelled like fir trees, but I got the dry leaves. We saw dry leaves, but we didn't smell them. So, that smells different from a fir tree.” 14, simulation, pos. 113*

“*I didn't smell anything now, or at least not as intensely as I would have expected in the forest.” 90, simulation, pos. 67*

“*But there was the smell, for example, the smell of the forest, and it was a bit too strong for me. At first it was almost like getting dizzy, the smell was so intense.” 67, simulation, pos. 19*

In the forest, smell only had positive connotations:

“*Here I found that the air is clear and you can feel that when you breathe, you want to breathe in and out deeply and that also helps to calm you down.” 82, forest, pos. 67*

The smell of damp earth, leaves, resins, and wood contributed to the calming and natural atmosphere of the forest:

“*The smell of earth, the smell of forest. Damp earth or damp leaves. Or of fir trees. That's also part of the calming effect.” 67, forest, pos. 59*

One participant made the comparison with aromatherapy, of which she is reminded by the diverse smells in the forest and noted a calming and refreshing effect. The smells have something “*refreshing”* and “*somehow something with clarity in it” (29, forest, pos. 47)*.

The olfactory sensory perception revived certain memories in some participants and evoked a feeling of security (*n* = 2). Similar to the acoustic stimuli, personal references to family or childhood were made, which reinforced the feeling of security. Furthermore, the variety of smells and the lively atmosphere in the forest is something that the participants experienced intensively.

##### 3.2.1.4 Visual perceptions

The simulated three-dimensional forest experience did not have the visual authenticity expected by the participants. The repetition of the video sequences meant that the simulation was not perceived as an equivalent alternative to the forest. The participants were unable to fully engage with the simulation and thus did not experience a comprehensive immersion.

“*I didn't feel like I was in the forest. For me it was like looking at an animated picture, but there was no comparison with the forest experience.” 95, simulation, pos. 7*

In addition, some participants were visually overstimulated, which led to overexertion and exhaustion (*n* = 11). This resulted in fatigue, which one participant described as “*pleasant,”* however, and indicated a complex compound effect arising from the sensory stimuli.

“*That's the question, because at the beginning I felt quite overwhelmed or simply flooded with stimuli. I would say that it was more like exhaustion, but it was actually also a pleasant feeling of being tired. So, as I said, at home I would have just gone to bed and closed my eyes.” 96, simulation, pos. 19*

Nevertheless, there was also positive feedback on visual perception: the viewing angle could be changed by moving the head, as well as birds or insects were flying through the picture.

“*Apart from that, what [...] I found quite nice were the animals, some of which flew quietly through the picture, they gave the whole thing a bit of life.” 36, simulation, pos. 15*

“*The immersion in the green and this closeness. That's something that feels real.” 29, simulation, pos. 43*

Overall, the participants described the simulation as less detailed and not realistic compared to the real forest. Improved color matching and image resolution in particular, as well as better adjustment options on the VR headset for people wearing glasses, were factors that participants considered necessary for an immersive simulation.

After the forest intervention, the participants described visual perceptions as the most central sensory experience in the forest. The interplay of colors, light, shadows, details, constancy, and changes in perspective generated a visually unique environment that helped participants relax, regenerate, and find inner peace:

“*And then the sun was shining. […] It was always like this, there were shadows everywhere on the ground and you could see so many details.” 28, forest, pos. 11–27*

The green color of nature and plants was perceived by many participants as particularly calming, as well as beneficial and conducive to inner peace (*n* = 21). Looking at leaves, trees and the surroundings led to immediate relaxation or relaxation that developed over the course of the stay and helped to let go of worries and everyday problems.

“*All the green was really good for me. I lay under a beautiful tree, looked up into the canopy of leaves the whole time and looked through the sky and it made me calmer and took away my worries [...] it was soothing to have this green color everywhere.” 95, forest, pos. 28–32*

Several participants (*n* = 7) reported that directing their gaze to distant elements, such as the canopy or the sky, was visually or mentally relaxing and helped them regain perspective (No. 95, 82, 12, 98, 32, 26, 39). One participant explicitly linked near-focus screen work to ocular strain and headache and described relief when regularly looking into the distance in the forest (39, forest, pos. 76).

The change from constantly looking at close objects to wide landscapes or the sky was not only perceived as relaxing for the eyes, but also as calming for the mind. Looking into the distance conveyed a sense of freedom and expanse that allows participants to detach themselves from the pressures and stresses of everyday life and opens the possibility of gaining new perspectives and finding inner peace.

In addition, the participants emphasized the importance of consciously observing the subtleties of natural phenomena that are often overlooked by individuals in their daily routines. The conscious observation of animals, plants or structures in nature, such as looking at beetles, butterflies, caterpillars or trees and plants, lead to a slowing down of thoughts away from the hectic pace of everyday life and a deeper immersion in nature. In addition, what they see triggers relaxation, satisfaction and wellbeing in the participants and is accompanied by an increased awareness of the beauty and complexity of nature, which enables a feeling of connection with nature and evokes a sense of wonder.

“*And what I also found somehow beautiful: If you concentrated on a place that actually looked very unspectacular, you saw that there was somehow a lot of small animals going on or you saw some spider threads where the sun got caught in them and then you noticed so many small things in detail.” 28, forest, pos. 27*

The size and age of the trees also contributed to participants' emotional and cognitive responses. The towering presence of ancient trees evoked a sense of perspective and scale, making personal problems appear smaller and less significant:

“*It creates such a relationship for me. [...] It makes me [...] so small when I think what he's already been through. And then I'm over 30 years old and he's already 100 and I don't know what exactly. It's like that, it puts things back into perspective.” 99, simulation, pos. 85–93*

“*And you're just a very small dot. [...] Yes, it sometimes puts a lot of things into perspective, so it also somehow gives you a perspective that you shouldn't always let everything get you down and that there is a solution for many things, for many problems, and that some things aren't so bad, even if they seem that way at the time. [...] Yes, also a bit of distance.” 82, forest, pos. 39–47*

#### 3.2.2 Psychological aspects

The emotional perception of the forest simulation showed a complex and differentiated spectrum of reactions, ranging from temporary relaxation and emotional security to pronounced irritation and frustration.

##### 3.2.2.1 Calmness/stress

**Figure 5:**
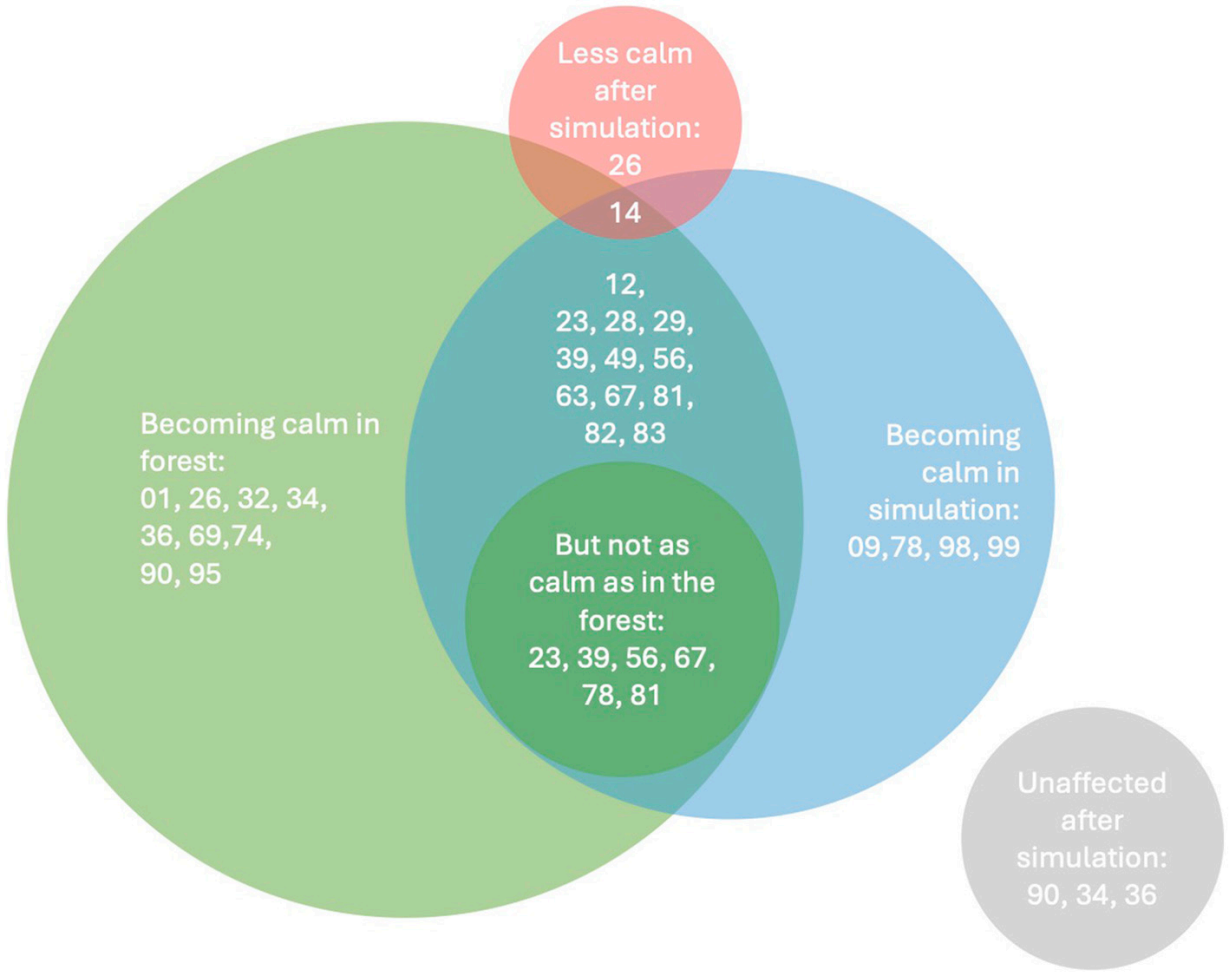
Venn diagram—Differences between forest and simulation. The Venn diagram (Figure 5) maps participant-level reports of becoming calm after each condition. The **left circle** lists individuals who reported calmness after the forest; the **right circle** lists those who became calm after the simulation; the overlap denotes participants who reported calmness in both settings. The small inner overlap features cases who explicitly stated that calmness in the simulation was weaker than in the forest. The gray set marks participants unaffected by the simulation, whereas the red set indicates those who felt less calm after the simulation. Taken together, the pattern aligns with our qualitative synthesis: calmness occurred in both environments but was broader and more pronounced following the real forest.

Particularly striking is the role of calmness and stress reduction, which was mentioned as the most frequent category in the simulation. Eighteen of twenty-four interviewed participants reported short-term relaxation, stress relief, or reduced tension during the simulation (*n* = 18/24 ≈ 75%). They reported a temporary reduction in stress and tension, but this did not reach the same depth as in a real stay in the forest (see Figure 5). The remaining six participants reported that the simulation did not help them reduce stress or reach calmness. Visual and auditory stimuli, in particular natural sounds such as birdsong and the gentle rustling of leaves, proved to be central to calming down.

“*But it was actually really good to just switch off for 40 min and now I'm calmer too.” 82, simulation, pos. 10*

A statement that shows that simulation can bring about short-term relaxation. However, it cannot replace the sensory depth of a real forest experience.

“*When I'm actually in the forest and can sit down, touch everything, really smell all the different bushes and such, [...] that the relaxation is actually greater in the forest.” 99, simulation, pos. 29*

Participants perceived the forest simulation as a place of retreat offering the participants the opportunity to take time for themselves without being disturbed.

“*There was simply no one around you [...] and so you had time for yourself, not to think about university at all.” 83, simulation, pos. 39*

The simulation was therefore also experienced as a place for reflection and the mental processing of personal feelings and emotions. Nevertheless, the assessment that it was a passive and unsustainable experience prevailed, as physical interaction and sensory diversity were lacking. One participant formulated this as follows:

“*In the real forest, I have the feeling that my body is participating [...]. Here it was more like looking at something, but it remains passive” 14, simulation, pos. 42*

A key aspect underlying the calming effect was the experience of time and the absence of external pressures. Several participants described how both forests (real and simulated) allowed them to slow down, escape the hectic pace of daily life, and reconnect with themselves:

“*I had the freedom here in the forest, the time. And somehow there's no pressure here.” 67, forest, pos. 27*

“*And when I go into the forest, I actively take the time. And in this timelessness, any tension within me subsides. And at the same time I free my thoughts a little from the more banal thoughts in everyday life.” 56, forest, pos. 67*

Participants used expressions such as “*getting out of the hectic pace,” “coming to myself,”* and “*finding my way back,”* often describing the forest as a return to something primal or original:

“*There's something so primal about it, to be in nature. [...] It feels so natural.” 95, forest, pos. 68*

*Part of the restorative effect also appeared to stem from the lack of expectations and obligations:*

“*Not having to do anything. [...] Nobody can want anything from me. [...] Relief somehow. So, it was really liberating.” 95, forest, pos. 40*

##### 3.2.2.2 Energy

Another key aspect of emotional perception was the change in the participants' energy levels. While some participants described increased alertness and an increased level of activity after the simulation (*n* = 3), others reported a state of relaxation that ranged all the way to fatigue (*n* = 13). This was a passive experience without physical activity, which did not allow for long-term revitalization or regeneration.

“*Right now, I'm very, very tired. I think halfway through I got very tired and then I just closed my eyes.” 96, simulation, pos. 7*

The simulation was not only related to the participants' own energy level, but also to the perception of a lack of energy and liveliness in the simulation. For example, that the trees did not emit any energy.

“*Well, it was nice to hear the birds. [...] Well, I missed the energy of the trees.” 14, simulation, pos. 16–17*

This observed state was complemented by a lack of vitality, so that no energy is transferred to the participant's own body. For one participant, this experience resulted in a “*disappointment for the senses and soul,”* the simulation was perceived as a “*lie”* and a false reality (26, simulation, pos. 15).

“*I was missing life. A bird flew from time to time [...] but it was dead. I somehow [...] didn't feel the energy on my body.” 14, forest, pos. 55*

The participants miss the authentic energy and liveliness of a real forest. The simulation was perceived as static and lifeless, which leads to a feeling of disappointment and does not contribute to an increase in personal energy levels.

In the real forest, participants reported that their energy levels increased. In particular, visual stimuli such as the play of light between the sun's rays and shadows, looking at plants and trees and the diversity of the surroundings were described as refreshing:

“*I find this constant change of sun, shadows and shapes very invigorating.” 28, simulation, pos. 27*

The perceived vitality of nature, combined with the opportunity to move around in the open space, increased general wellbeing and often led to a feeling of alertness and strength:

“*Very relaxed and really powerful and full of energy. I would love to run up a mountain or go for a longer hike.” 63, forest, pos. 63–68*

“*I feel really firmly in the saddle right now. Secure, even. And then also powerful. And that I think: Yes, no matter what the day brings today. [...] I can handle it well.” 98, forest, pos. 57–61*

However, feelings of tiredness were also occasionally described. These sensations resulted primarily from the release of inner tension that caused a kind of regenerative tiredness:

“*I noticed that I started yawning a lot. So much so that my eyes were watering too. I became so calm and invigorated inside.” 98, forest, pos. 19*

“*Once this calmness, that everything vibrates in its own rhythm. [...] You can't see it, you can't hear it - but you can hear it in the rustling, soft rustling of leaves or bark, branches and birdsong. Or a dragonfly flying by. Very quiet, but also audible. This feeling of being connected.” 26, forest, pos. 11*

##### 3.2.2.3 Feeling protected/security

Participants also reported feeling protected and secure, which was another important element of their emotional response to the simulation. While some participants perceived the simulation as a protected retreat, many expressed that the lack of the natural environment significantly limited their sense of calm and protection (*n* = 3 vs. *n* = 5). Furthermore, the perception of being protected and feeling safe in the forest simulation was strongly dependent on the authenticity and the quality of the virtual environment experienced by the respective participant. Physical elements in the video such as hills offered a feeling of protection, while natural dynamics such as wind movements strengthened the emotional connection to the environment. However, the simulation did not achieve the depth of emotional resonance necessary for a lasting feeling of security. Therefore, the simulation was rated neutral from the participants in this context and does not strengthen the feeling of security. Disturbing factors, such as the presence or noise of other people, showed to impair feelings of security.

“*My condition was influenced by the thoughts themselves. The simulation didn't stress me out or make me feel insecure or worried at all. The forest simulation didn't have this strong influence on me, where I can feel safe or where I get clarity in my thoughts.” 67, simulation, pos. 37*

Nine participants perceived the forest as a place of protection and security. The natural structure of the forest with its tall trees, dense canopy and hidden, sheltered areas supported a profound sense of security and emotional warmth:

“*It's warm, it's calm, it's harmony. [...] Full confidence. I am part of the forest, so to speak.” 14, forest, pos. 31*–*35*

The reduced presence of urban stimuli and human noises supported the perception of a safe retreat that enabled undisturbed reflection and processing of personal emotions. The atmosphere was perceived as good and “*pleasant”*:

“*I felt comfortable in there” 29, forest, pos. 63*

However, there were isolated mentions of insecurities or fears, particularly among female participants, who might feel less safe outside the study conditions if they were alone in the forest.

“*You don't feel so safe alone in the forest after all.” 32, forest, pos. 39*

Additionally, some participants reported moments of further discomfort, such as feeling cold. Nevertheless, these ambivalent moments were typically transient and did not overshadow the overall restorative effect of the forest.

##### 3.2.2.4 Freedom vs. restriction

Feelings of confinement were widespread during simulation. Wearing the VR headset often led to sensations of restriction and oppression, sometimes resulting in stress, anxiety, or even panic:

“*The headset was just heavy and oppressive on the head.” 69, simulation, pos. 7*

“*When we did the other study on the chair, I felt so cramped and squeezed in and couldn't move so freely.” 98, forest, pos. 23*

Disorientation and an oppressive feeling also occurred, as the participants were unable to react adequately to sounds in the real environment, which sometimes drown out the sounds of the simulation. The discrepancy between the virtual viewing window and the participants' awareness of the actual environment has been shown to induce stress and anxiety, and in some cases, to evoke panic (*n* = 3).

“*To be honest, I almost panicked a little at the beginning. It was such a stressful feeling inside me. [...] I found it very oppressive that I couldn't take my glasses off [...] if there was a noise somewhere, I automatically looked at it, wanted to orientate myself. But I only see the forest and not the real surroundings.” 95, simulation, pos. 11*

In the real forest the topic of freedom vs. restriction played an important positive role. Nineteen participants described the forest as liberating and felt a reduction in social pressure and obligations:

“*I had freedom here in the forest, the time. And there is no pressure here.” 67, forest, pos. 27*

The deliberate absence of technological distractions such as smartphones increased the feeling of freedom and promoted a conscious experience of the present moment. Participants found the physical freedom of movement in the forest particularly pleasant, clearly contrasting it with the restriction and lack of mobility they experienced in urban environments or simulations. Although solitude was valued by many (*n* = 9), some participants reflected on the absence of social interaction, occasionally experiencing loneliness or wishing to share the experience with others (*n* = 3). This emphasizes the nuanced role of social context in shaping the restorative potential of the forest.

“*This feeling of not having to do anything. [...] Now I'm just with myself and nobody wants anything from me. No cell phones ringing, no tasks to do. [...] It felt really liberating.” 95, forest, pos. 36–40*

Being confronted with oneself, without other people and without a mobile phone, led some participants to reflect more deeply and organize their thoughts:

“*[...] that I am repetitive in some thoughts, constricted. Experiencing nature can be liberating. That I can take an outside perspective on my thinking.” 34, forest, pos. 98*

“*I found it very good that I now had the opportunity to be alone with myself for a bit, without any stimuli, people or whatever. And so I could reflect on what had happened and all sorts of things. [...] With myself, but really alone. And not only without people, without a mobile phone, but also without the city. So really all alone.” 67, forest, pos. 7*–*11*

Detaching oneself from problems in the forest environment reduced feelings of constriction and promoted a sense of openness. Two participants reflected this as follows as stated below:

“*The thoughts and the worries. And now I can feel that there is a bit more space, a bit more options.” 95, forest, pos. 44*

“*From being very held and controlled to a more detached state of not being so completely head heavy.” 29, forest, pos. 7*

##### 3.2.2.5 Sadness/happiness

Participants in the forest also reported feelings of sadness and happiness. The forest offered them a space to pause, experience and reflect on their emotions more intensively:

“*Contentment, but also a little sadness, because I'm generally very melancholy at the moment and don't find much time to think about it.” 28, forest, pos. 43*

At the same time, positive moods could be promoted by the calm atmosphere of the forest, which triggered feelings of happiness:

“*I was a bit more joyful at the beginning. Just being in the forest, feeling this peace.” 34, forest, pos. 33*

The strong sensory perception of the natural environment intensified the feelings and led to both joy and melancholy being perceived more consciously.

In summary, the real forest evoked strong positive emotions and feelings in the participants due to its complex, multi-sensory environment and the opportunity to interact directly with nature. The various dimensions of the experience illustrate the importance of real experiences of nature for the emotional and psychological wellbeing of the participants. In addition, participants reported that the forest not only enabled short-term relaxation but could also have a lasting impact on the general perception of stress, psychological wellbeing and resilience.

### 3.3 Activities

During their stay in the forest, the participants reported engaging in or imagining a variety of activities that enhanced their sensory and emotional experiences, consistent with findings from previous semi-structured interviews in natural environments (Oomen-Welke et al., 2023). These (imagined) activities consisted in slow walking, hiking, running, cycling, or jogging to relieve stress, as well as periods of rest and intentional relaxation. The participants frequently chose to sit or lie down (e.g., 95, forest, pos. 28–32), observe the sky through the treetops (Oomen-Welke et al., 2023), listen to birds (e.g., 26, forest, pos. 11), and consciously attend to acoustic, tactile, and olfactory stimuli (e.g., 67, forest, pos. 59). They also physically explored natural elements such as moss, bark, or leaves (e.g., 39, forest, pos. 79–83). Several participants recalled childhood memories of playing, building huts, or balancing on fallen trees. The majority of participants expressed a preference for fully immersing themselves in the multisensory forest environment, deliberately avoiding distractions such as music to maximize their perceptual experience of the environment. Conversely, the participants expressed criticism of the simulation, citing a lack of opportunities for movement and tactile interaction.

### 3.4 Ideal natural and simulated environments

Participants consistently described an ideal forest as lively and diverse, characterized by a rich variety of plants and animals, as well as natural features such as watercourses or ponds. One participant remarked,

“*[In the forest], I can concentrate better on nature because it's more varied. You don't see just this one picture. (...) You can maybe walk around a bit and see something new every now and then” 36, simulation, pos. 23*

Participants considered this sense of liveliness and diversity essential for fostering engagement and relaxation, as well as for enabling them to feel like active participants rather than passive observers within the environment.

The ideal natural environment for maximum relaxation and stress reduction included bodies of water, such as calm lakes or gently flowing streams, which participants perceived as visually calming and providing relaxing sounds. They emphasized that a diverse range of healthy vegetation, such as mixed forests containing deciduous trees, mossy ground, ferns and aromatic conifers, was particularly beneficial. Small, natural footpaths were seen as supporting harmonious orientation without disturbing the naturalness of the environment. Participants indicated that the surroundings should be free of human noises, instead offering the discreet chirping of birds, the gentle rippling of water, and the soft rustling of leaves in the wind. The presence of visible, unobtrusive animals, such as squirrels, birds, and deer, were described as enhancing the feeling of liveliness. Pleasantly warm, slightly temperate air was also considered important. Participants described a varied landscape with alternating sparse and dense areas, clearings, and waterholes, as well as minimal visible human influence and natural deadwood, as reinforcing the feeling of security and originality, thereby leading to maximum relaxation.

In contrast, participants suggested that the ideal simulation of a forest environment should include elements that make the experience more authentic and relaxing: a realistic visual representation with high image resolution and natural, non-oversaturated coloring, especially of greens and the sky. “*The first thing would be to have a sharper image, like you would see normally. In the middle it was sharp, but above and below it was blurry” (95, simulation, pos. 108); “The green didn't look authentic, it was oversaturated” (67, simulation, pos. 117–121)*. They suggested that longer, non-repetitive video sequences would improve the sense of immersion: “*Maybe a longer video, so the repetitions don't happen so often” (95, simulation, pos. 112)*.

They recommended integrating moving elements, such as swaying leaves or birds flying by, and proposed that a longer video recording without conspicuous repetitions could improve the experience. Participants repeatedly emphasized the importance of freedom of movement and flexible, comfortable seating, such as the ability to move a few steps, lie down, or choose alternative seating like rotating chairs or yoga mats:

“*I would have liked to be able to move a few steps or lie down. On the chairs, I didn't really feel like I was in the forest” 28, simulation, pos. 127*

They also valued the option to turn or change position easily:

“*Either something rotating so I can turn around, or something without armrests, so I can also sit sideways” 63, simulation, pos. 37–39*

Participants expressed that significantly improved freedom of movement would be desirable and the ability to move a few steps around the room, sit down, or lie down could make the experience more authentic. More suitable seating options, such as swiveling chairs without backrests, comfortable armchairs, or yoga mats, were also considered advantageous.

Participants further suggested that a more authentic environment could be achieved if the simulation took place outdoors or at least in a well-ventilated room to ensure fresh air and an appropriate temperature (between 15 and 21 degrees Celsius). Multisensory stimulation was also considered essential. Participants reported the need for authentic, diverse natural sounds, subtle but present scents, preferably from natural sources like wood chips rather than artificial diffusers, and haptic elements such as moss or wood to enhance immersion:

“*I would use wood chips instead of a diffuser, because that smells more natural” 14, forest, pos. 121*

They also noted that the essential oils used in the simulation were only noticeable at first and suggested stronger or more persistent natural scents:

“*If you want to smell something, you should make sure you can actually smell it” 39, simulation, pos. 123*

Participants believed that haptic and sensory elements could further enhance perception, for example, by using natural wood chips instead of a diffuser, stronger odors from real plants, and subtle but varied natural sounds.

They recommended that the simulation should also take place in a secluded, preferably noise-insulated room to minimize distractions caused by outside noise or technical problems. A quiet, well-ventilated, and isolated room was seen as crucial to avoid external disturbances:

“*A room that is a bit more secluded or soundproofed, so you don't hear outside noises. That would definitely help” 56, simulation, pos. 111*

Furthermore, the social context was important. Participants preferred being alone or only with familiar people and disliked disturbances from others:

“*You don't see the people, but you hear them somehow. That makes a difference” 96, simulation, pos. 41*

Alternatively, participants mentioned that a simulation of a quiet bay with a sandy beach, turquoise-colored water, and crashing waves could also contribute to relaxation. Overall, they emphasized that the experience should be multimodal in order to create a comprehensive and authentic nature experience.

Despite these possibilities, participants acknowledged certain limitations to achieving a truly ideal simulation. Some sensory and emotional aspects, such as the perception of wind, complex natural scents, and the deep sense of freedom and connection experienced in real nature, were considered difficult to fully replicate:

“*I don't think you can really recreate the wind. You perceive it in five dimensions, I don't think that works to recreate it” 23, forest, pos. 71*

Others described the simulation as a valuable alternative to gray buildings, but not a full substitute for the forest: “*For me it's an alternative to a gray wall, but not to the forest” (95, simulation, pos. 108); “It didn't feel real” (78, simulation, pos. 47)*. Nevertheless, many participants agreed that with further technical and sensory improvements, virtual simulations could become a highly realistic and beneficial supplement, especially when access to real nature is limited (*n* = 17).

Moreover, participants described the forest's self-sufficiency, appreciating its existence independent of human needs or intervention:

“*What do you like about the forest?” – “That it exists on its own. Without needing anything from people. Without having to give anything to people. It is simply an entity unto itself.” 67, forest, pos. 81–83*

## 4 Discussion

In this study, we investigated the effects of a real forest stay vs. experiencing a video-based forest environment on stress perception and psychological wellbeing, specifically focusing on sensory perceptions and emotional responses in HSP. Our findings support the hypothesis that significant differences exist between these two environments, with the real forest clearly demonstrating stronger positive effects on participants across the evaluated dimensions. These results align with previous research suggesting that authentic sensory experiences in nature mediate relaxation and has a restorative effect (Oomen-Welke et al., 2023). Furthermore, our findings corroborate theoretical frameworks suggesting that disparities between authentic and simulated natural environments substantially influence psychological and physiological responses (Ulrich, 1983; Kaplan, 1995; Slater and Sanchez-Vives, 2016).

There are several studies in the field of forest simulation which have different research approaches. Controlled studies indicate that simulated forest environments (VR, multisensory 360° VR, and digital twins) reliably yield short-term reductions in stress and improvements in mood. Across these experiments, control conditions varied: randomized comparisons contrasted realistic vs. “dreamlike” VR forests with identical terrain, interactions, and sounds (Alyan et al., 2021), Masters et al. (2025)) included a non-VR rest control (eyes closed) in addition to high- vs. low-realism VR nature. Greater ecological realism and multisensory enrichment are associated with larger effects (larger decreases in heart rate and skin conductance in realistic vs. dreamlike scenes). Olfactory stimulants further enhance relaxation (Wang et al., 2019; Alyan et al., 2021; Clemente et al., 2024; Masters et al., 2025). Digital twin pilot studies achieve relaxation outcomes approaching those of real forest exposure without consistent superiority (Hejtmánek et al., 2022), and standardized protocols (VR plus phytoncide delivery) have been proposed to improve reproducibility (Ross and Jones, 2022). Our quantitative analyses showed consistently larger effect sizes in the real forest than in the 360° VR simulation.

Participants consistently reported in the quantitative questionnaires as well as in the interviews significant physical, mental, and emotional relaxation in the real forest, confirming the role of multisensory stimuli such as natural sounds, fresh air, tactile interactions, and visual impressions. The forest's green colors, dynamic environmental conditions, and authentic natural elements fostered an intense sense of security and emotional warmth, significantly enhancing overall relaxation and stress reduction. In contrast, the video-based forest simulation, despite successfully inducing temporary calmness through auditory and visual stimuli, notably lacked authenticity and sensory diversity. Participants particularly missed haptic interactions and realistic scents, and they frequently criticized technical limitations such as visual blur and repetitive video loops, which significantly impaired immersion and relaxation. Also, movements are difficult to simulate, especially when participants wear head-mounted displays. This restriction of physical movement is particularly relevant from the perspective of self-determination theory, which identifies autonomy (the sense of acting freely) as a fundamental psychological need linked to wellbeing (Ryan and Deci, 2000). For an overview of the differences in the perception of sensory modalities, see Table 5.

**Table 5:** Sensory modality comparison: authentic forest vs. video-based forest simulation.

Sensory modality	Forest environment	Video-based forest simulation
Haptic interaction	Natural temperature gradients, wind on skin, forest floor texture, humidity	Absent (controlled room temperature 21–30 °C, seated position)
Acoustic perception	Spatial 3D soundscape, dynamic wind/birdsong, distance-dependent attenuation	Recorded forest sounds synchronized with video, looped audio sequence
Olfactory perceptions	Natural forest air (soil, vegetation, phytoncides), dynamic intensity variation	Nebulized spruce needle essential oil, constant artificial diffusion
Visual perceptions	Natural stereoscopic depth, unlimited field of view, dynamic light/shadow, authentic color spectrum	Monoscopic 360° video (5.7K), head-tracked viewing, 6.5-min looped sequence

Potential bias factors include social contacts and physical activity. We minimized social contacts in both groups and instructed forest participants to focus on sensory perception rather than physical exercise. Simulation participants also walked approximately 400m when arriving by public transport. Both conditions had comparable baseline measurements unaffected by prior physical activity (blood pressure, heart rate, STAI, BMS). While we cannot completely exclude that our results are biased by these factors, we regard such bias as unlikely given the balanced baseline characteristics. Another potential limitation is the erroneous allocation of two participants to the wrong intervention sequence, which occurred because they did not read the allocation email in time. Following intention-to-treat principles, we retained these participants in the sequence they actually received, resulting in final group sizes of 27 and 22 rather than the randomized 25 and 24. As baseline parameters remained similar between groups, we regard this deviation as minor.

We were unable to simulate the odor of the forest floor or the fresh air sufficiently, which was perceived as refreshing by the participants. It seems that the sense of smell tends to become accustomed to odors in a room, possibly because they remain constant (Sinding et al., 2017; Schreiner et al., 2020; Mignot et al., 2022). In the forest, the wind changes and modulates odors. Due to the constant change, odors are always perceived anew without any adaption (Bentley et al., 2023).

The limited sensory authenticity of the simulation revealed critical deficits, indicating that the real forest environment provides irreplaceable benefits related to psychological safety, emotional depth, and stress alleviation. The reduced restorative effects can be understood through presence theory. Multisensory integration is essential for the sense of “being there” and the absence of tactile, kinesthetic, and vestibular input in our simulation setup likely compromised this presence, particularly for highly sensitive persons their enhanced sensory processing capabilities (Aron and Aron, 1997; Slater and Wilbur, 1997). These may amplify the differential impact of authentic vs. simulated natural environments. Therefore, authentic forest experiences constitute an essential resource for psychological wellbeing and stress management in this population.

Notably, participants valued the simulation as a convenient and brief opportunity for short-term relaxation and reflection. They recognized its practical value in helping them to integrate moments of calmness into their daily routines, particularly when access to a real forest is limited. Nevertheless, participants did not experience persistent emotional or energetic benefits from the simulation, emphasizing the necessity of genuine natural interactions for prolonged stress relief and revitalization.

The divergent reports regarding participants' energy levels during the simulation suggest that individual differences exist with regard to physiological and psychological responsiveness to sensory stimuli. These variations may be attributable to baseline conditions (e.g., age, sex), personal expectations, or the individual threshold for sensory immersion (Browning et al., 2020; Spano et al., 2023). Future research should examine these individual factors more closely to optimize simulation environments.

Several female participants anticipated feeling unsafe if visiting the forest alone. This aligns with socio-spatial scholarship showing that women's fear is shaped by gendered norms, perceived risks of interpersonal violence, and the affordances of particular landscapes rather than individual disposition alone (Valentine, 1989; Pain, 1997). However, the restorative effect of the forest typically overshadowed these transient moments of ambivalence.

Based on participant feedback, we found several key points for improving the simulation. One key recommendation is to record the simulation video for the full duration of the intervention, thereby avoiding repetitive loops and enhancing immersion. Capturing the dynamic play of light and shadow in the video could further increase visual interest and authenticity. Additionally, simulating haptic feedback and providing a quiet, well air-conditioned, and sound-insulated room would improve comfort and reduce external distractions. The more different sensory factors interact synergistically leading toward fully interactive virtual realities, the stronger is the effect of connectedness and security, ultimately leading to relaxation and self-centring which aligns with other literature (Slater and Sanchez-Vives, 2016; Suseno and Hastjarjo, 2023; Ascone et al., 2025).

Overall, the findings underline the intrinsic value and irreplaceable quality of authentic multisensory experiences provided by natural forests, emphasizing the limitations of current technological approaches to a simulation of multimodal natural environments. While technical improvements could enhance future simulations, achieving equivalence with real forest experiences, particularly regarding sensory diversity, haptic interactions, and authentic environmental dynamics, remains a significant challenge. Thus, real forest settings should continue to be prioritized in health-promoting interventions aimed at stress reduction and psychological wellbeing, especially for HSP.

## 5 Conclusion

Real forest is more effective in reducing stress and inducing wellbeing and positive feelings than a video-based forest simulation in HSP. Creating a realistic forest simulation is technically complex but may be optimized especially regarding haptic, olfactory, visual stimuli. A simulation of a natural environment should provide the possibility to move while experiencing the simulation.

## Data Availability

The datasets generated during this study are not publicly available due to ethical and privacy restrictions. Participants provided written informed consent for study participation but did not consent to public data sharing. As stated in the participant information, personal data would not be shared with third parties. The qualitative interview transcripts contain sensitive information that could potentially identify participants despite anonymization procedures. Quantitative data may be made available by the corresponding author upon reasonable request. Access is subject to a formal data sharing agreement to ensure participant confidentiality and compliance with the ethics approval and participant consent agreements. Requests will be evaluated on a case-by-case basis. The semi-structured interview guide is available as Table S3 in Supplementary materials.
