# Supplementary material for: Spending time in a forest vs. a virtual forest simulation: qualitative and quantitative effects on stress perception and psychological wellbeing in a randomized cross-over trial of highly sensitive persons
Source: Front Psychol. 2026 Apr 8;17:1707766. doi: 10.3389/fpsyg.2026.1707766 (PMC13110857; doi:10.3389/fpsyg.2026.1707766)
Supplement: Supplementary file 1 [file Data_Sheet_1.docx]

# Appendix - Code definitions

## Forest

Definition

This code marks all sections in which the forest is discussed. This makes it easier to distinguish between them in the subsequent analysis, in which this code can be combined with the other codes and only the relevant passages are shown.

## Simulation

Definition

This code marks all sections in which the forest simulation is discussed. This makes it easier to distinguish between them in the subsequent analysis, in which this code can be combined with the other codes and only the relevant passages are shown.

## Sensory perception

Definition

Refers to the subjective experiences and descriptions of the participants with regard to their sensory perceptions (sight, hearing, touch, smell, taste) during their stays in the forest or in the forest simulation, which are evoked by the environment or specific events. Related sections are always chosen, with the selection of the code being based on the main sensory perception.

## Emotions, Feelings and Thoughts

Definition

Passages that evoke emotions, feelings and sensations that can be assigned to one or more subcategories in terms of their quality. In addition, these are passages in which very complex impressions are conveyed which, due to their complexity, cannot be divided into individual categories without destroying the overall impression of the passage.

## Energy

Definition

The participant describes a subjective feeling of energy that is characterized by the fact that you can do more afterwards or simply feel more alert, fitter, etc. The feeling of energy can be experienced physically or mentally, paying attention to whether it is associated with certain activities or sensory impressions or whether it changes over the course of the stay.

## Feeling protected / Security

Definition

Refers to the subjective feeling of safety, protection and security that participants experience during their stay in the forest or forest simulation. This feeling can be evoked by physical aspects of the environment (such as dense trees, surrounding vegetation) or by emotional states (such as inner peace, absence of threat).

## Diversity

Definition

Records the participants' perception and description of the variety and diversity of sensory impressions, natural elements (plants, animals, landscape) or the overall atmosphere during their stay in the forest or forest simulation. The diversity or richness of variety of the environment is described, regardless of whether it is rated positively, negatively or neutrally.

## Liveliness / Being alive

Definition

Captures participants' subjective experiences and descriptions of the feeling of liveliness, dynamism and energy they perceive while in the forest or forest simulation. It is about how the environment is experienced as lively, active and full of energy.

In the latter case, an overlap with the energy code is possible.

## Sadness / Happiness

Definition

Records the subjective experiences and descriptions of the participants that relate to the feeling of sadness, depression, melancholy or emotional pain that they experience during their stay in the forest or in the forest simulation. As a polarity to this, the code happiness captures the opposite feeling in the emotional spectrum.

## Calmness / Stress

Definition

Calmness captures the subjective experiences and descriptions of the participants, which relate to the feeling of calm, relaxation, serenity and inner peace that they experience during their stay in the forest or in the forest simulation. It is about how the environment promotes a reduction in stress, tension and anxiety and contributes to a state of balance.

Stress records the subjective experiences and descriptions of the participants that relate to the feeling of stress, tension, pressure or excessive demands that they experience during their stay in the forest or in the forest simulation or that existed before the stay and was reduced by it. It is about how the environment is perceived as stressful, demanding or overwhelming.

## Surprise

Definition

Captures participants' subjective experiences and descriptions of the sense of surprise, wonder or amazement they experience while in the forest or forest simulation. It is about how unexpected or unusual events, impressions or encounters in the environment evoke an emotional reaction of astonishment or wonder.

## Fear

Definition

Captures participants' subjective experiences and descriptions of the feelings of anxiety, fear, apprehension or uncertainty they experience while in the forest or forest simulation. It is about how the environment is perceived as threatening, frightening or unsafe and which specific triggers or amplifiers for these emotions can be identified.

As this is a strong emotion that can have far-reaching consequences for the evaluation, this code is retained despite the rare text passages that refer to it.

## Freedom vs. Restriction

Definition

Records the subjective experiences and descriptions of the participants with regard to the feeling of freedom or confinement in the two environments (forest and forest simulation), and how these sensations are related to liberating or anxiety-relieving feelings. It is about how the spatial expanse or confinement of the environment influences the emotional experience and whether this leads to feelings of fear or liberation.

Polarities:

**Freedom:** expansiveness, openness, boundlessness, possibility of movement and exploration, feeling of independence and self-determination. Can be associated with positive emotions such as joy, relief and self-confidence, but can also trigger fear or excessive demands in some people.

**Restriction:** Limitation, restriction, confinement, feeling of control or surveillance, restricted freedom of movement. Can be associated with negative emotions such as fear, anxiety, frustration or loss of control, but can also give some people a feeling of safety or security.

Difference to security:

While “security” describes a feeling of protection and safety that can often be associated with a comfortable confinement or enclosure, "confinement" in this context refers more to a negative, frightening or restrictive experience of limitation.

## Satisfaction

Definition

Captures participants' subjective experiences and descriptions related to the sense of satisfaction, well-being, fulfilment or joy they experience while in the forest or forest simulation. It is about how the environment creates positive emotions and a general feeling of satisfaction. The reference is also to themselves or to the situation.
