# Supplementary material for: Spending time in a forest vs. a virtual forest simulation: qualitative and quantitative effects on stress perception and psychological wellbeing in a randomized cross-over trial of highly sensitive persons
Source: Front Psychol. 2026 Apr 8;17:1707766. doi: 10.3389/fpsyg.2026.1707766 (PMC13110857; doi:10.3389/fpsyg.2026.1707766)
Supplement: Supplementary file 2 [file Data_Sheet_2.docx]

Supplementary Material

# Supplementary Figures and Tables

## Qualitative Analysis

### Psychological Aspects

**Diversity**

The evaluation of the diversity within the simulation was mainly critical, the impression of a static, less interactive environment prevailed, even though some participants could adapt.

“And then I tried to adjust to it a bit and imagine that I was really in the forest and took a deep breath and just let it all sink in. And yes, it was amazingly real.” 49, simulation, pos. 11

Only one participant found that the presentation of the simulation was rich in detail and could certainly be perceived as varied.

“There was undergrowth, you could look at the bushes, foliage, some birds that flew off again in the distance. There were also big trees with big treetops. You could look up into the air. It was actually quite varied.” 90, simulation, pos. 107

They experienced a lack of stimulation of their own creativity and mental activation. “So there wasn't this stimulation of my own creativity or inner movement, but rather oh God, mindless, monotonous and oh no, not another 6 minutes.” 26, simulation, pos. 71

In the forest, diversity was experienced in the form of constant change, intense sensory impressions and the presence of animals and plants. *“Constant change. [...] And yet somehow there is always order in it. A stillness. Or simply a liveliness” (29, forest, pos. 91).* This dynamic interplay of nature, perceived as harmonious, led to a heightened perception of the moment and an increased awareness of one's own existence within a larger whole: *“Sometimes when I'm traveling, I have the feeling that you realize how much life is happening in this world at the same time. You are just a small dot.” (82, forest, pos. 39).* Experiencing natural cycles and the immediate proximity to life fostered a feeling of connectedness and diversity of life. Participants also described the forest as possessing its own rhythm, which encouraged them to slow down, synchronize with natural cycles, and experience time differently from their everyday routines.

The diversity of the forest was perceived and appreciated by many of the participants as meaningful for their stay (n = 13). The variety of trees as well as water elements were mentioned by nine participants, seven stated the importance of the variety of forest ground and five about the presence of bushes and dead wood. The diverse sensory impressions, the continuous change in the environment and the rich visual details were cited as key factors for a lively yet calming atmosphere. The influence of the weather in particular, especially the presence of wind, played an important role in this experimental setting and was sometimes even missed when it was not present.

“I also noticed once again how many qualities interact at the same time. Light, air, earth.” 26, forest, pos. 11

“And then the wind always came. It was wonderful.” 98, forest, pos. 11

## Tables

Table S1: Randomization and allocation of qualitative interviews

| Intervention Day | Time slot | Total participants (n) | First forest (n) | First simulation (n) |
| --- | --- | --- | --- | --- |
| Day 1 | 1 | 10 | 6 | 4 |
|  | 2 | 9 | 6 | 3 |
|  | 3 | 10 | 5 | 5 |
| Day 2 | 4 | 8 | 4 | 4 |
| Day 3 | 5 | 7 | 3 | 4 |
|  | 6 | 4 | 2 | 2 |
| Subtotal 1^st^ intervention | 6 time slots | 48 | 26 | 22 |
|  |  |  |  |  |
| Washout period | 7 days |  | Simulation* | Forest* |
| Day 7 | 1 | 9 | 5 | 4 |
|  | 2 | 7 | 4 | 3 |
|  | 3 | 12 | 7 | 5 |
| Day 8 | 4 | 8 | 4 | 4 |
| Day 9 | 5 | 6 | 3 | 3 |
|  | 6 | 4 | 2 | 2 |
| Subtotal 2^nd^ intervention | 6 time slots | 46 | 25† | 21† |
| Dropouts (period 1→2) |  | 2 (4.3%) | 1 | 1 |

*Cross-over: Groups received alternate condition in period 2. Maximum participants per time slot = 12 to minimize disturbance, minimal group size per time slot = 2. † Cross-over: “First forest” group received simulation in period 2, “First simulation“ received forest in period 2.

Table S1 presents the allocation of all participants across both interventions. Two interviews were recorded per intervention site and time slot resulting 48 interviews. 20 participants were interviewed after both interventions. One participant from the “first forest” group was replaced in the simulation by an alternative interviewee in the second intervention and one participant from the “first simulation” group was replaced by an alternative interviewee in the second intervention.

Table S2: Intercoder Agreement Main Codes

| Code | Agreement | Disagreement | Total | Percentage of agreement between coders |
| --- | --- | --- | --- | --- |
| Calmness / Stress | 43 | 3 | 46 | 94 % |
| Haptic Interaction | 41 | 3 | 44 | 93 % |
| Acoustic Perception | 35 | 5 | 40 | 88 % |
| Visual Perceptions | 36 | 11 | 47 | 77 % |
| Olfactory Perceptions | 30 | 11 | 41 | 73 % |
| Energy | 19 | 7 | 26 | 73 % |
| Feeling protected / Security | 12 | 8 | 20 | 60 % |
| Freedom vs. Restriction | 7 | 13 | 20 | 35 % |
| Total | 223 | 61 | 284 | 79 % |

## Photos


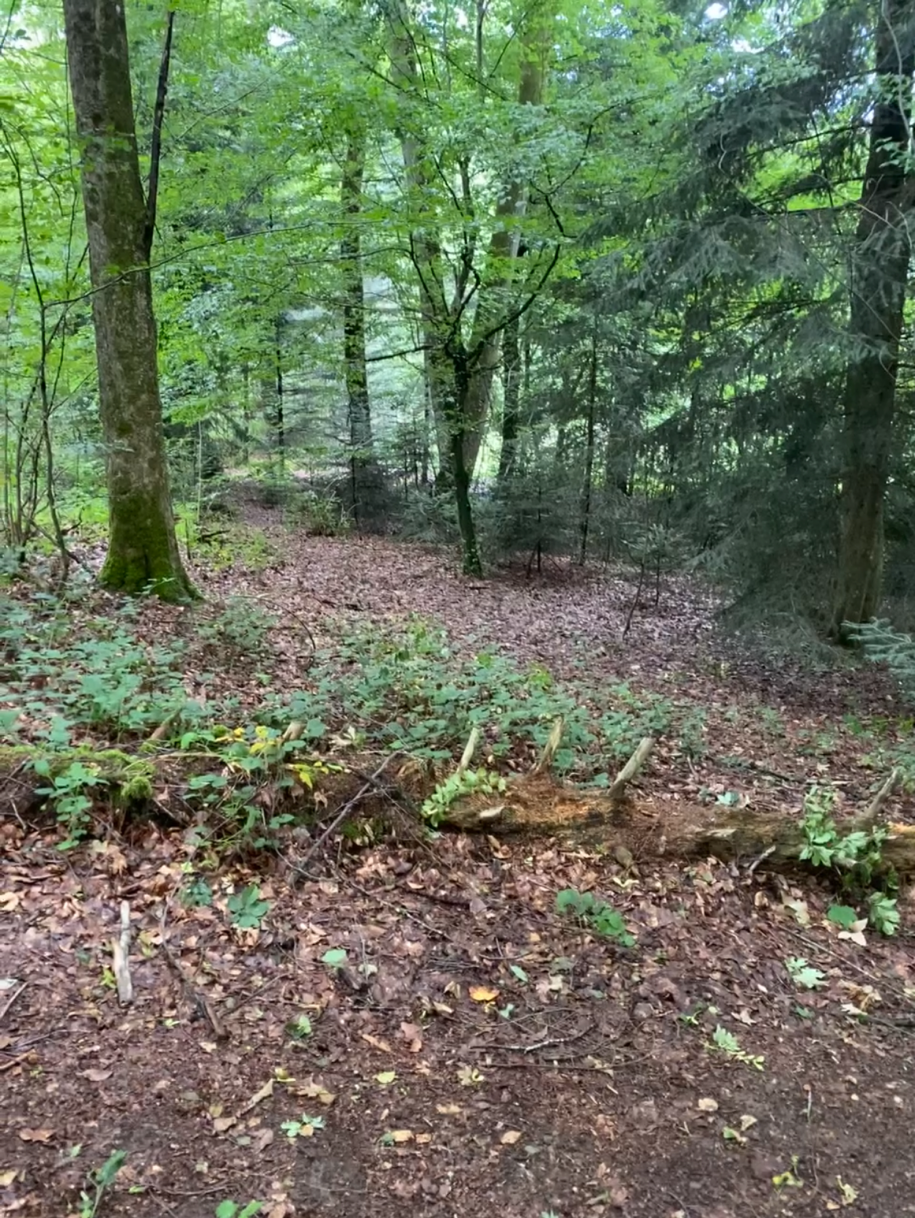
This picture is showing the typical environment of the intervention site in the forest.

Figure S1: Photo of the forest intervention site.

**Table S3: Semi-Structured Interview Questions**

| Question No. | Main Question | Pay attention to the following aspects | Follow-up Questions | Keep telling Questions |
| --- | --- | --- | --- | --- |
| **1** | **Initial Experience** |  |  | Please tell me about it. What else comes to your mind?  I would like to be able to imagine it, so please tell me more about it.  How do you experience it?  Can you tell me more about it?  And then?  Can you elaborate on that, please?  Can you be more specific? Have we forgotten anything? |
| 1.1 | You have just spent 40 minutes in the forest/forest simulation. How did you experience it? | Which aspects of the forest/forest simulation did you particularly notice? | What were your feelings and sensations during the stay?  To what extent would you say this has something to do with the forest/forest simulation?  What did you think about during your stay in the forest/election simulation?  What in particularly appealed to you? |  |
| **2** | **Post-Intervention Effects** |  |  |  |
| 2.1 | How are you feeling now? | After-effects: Perceptions in current situation | What are you taking with you right now?  Which aftereffects has the stay in the forest/forest simulation on you? |  |
| **3** | **Comparative Experience** |  |  |  |
| 3.1 | What appeals to you in general about the forest / forest simulation? | What does the person like about the forest/forest simulation and what do they dislike?  Values, assessments, associations | If you are going to the forest in your free time, why do you do it? How often do you do that?  What do you associate with the forest?  How do you feel about what you see here? (air quality, sounds, smell, haptics)  What does that trigger in you? |  |
| **4** | **Ideal Nature Experience** |  |  |  |
| 4.1 | Wonder question: What would the ideal forest/ forest simulation has to look like for you to feel particularly comfortable there? | Natural conditions that have positive effects | What is the ideal natural environment from which you can relax?  If you could wish for a place, where would you like to relax completely, what would it look like? |  |
